# Processing efficiency in pediatric cancer survivors: A review and operationalization for outcomes research and clinical utility

**DOI:** 10.1002/brb3.2809

**Published:** 2022-11-03

**Authors:** Julie A. Trapani, Donna L. Murdaugh

**Affiliations:** ^1^ Department of Psychology University of Alabama at Birmingham Birmingham Alabama; ^2^ Department of Pediatrics University of Alabama at Birmingham Birmingham Alabama

**Keywords:** cognitive late effects, neuropsychology, pediatric cancer survivors, processing efficiency

## Abstract

**Objective:**

Childhood cancer and cancer‐related treatments disrupt brain development and maturation, placing survivors at risk for cognitive late effects. Given that assessment tools vary widely across researchers and clinicians, it has been daunting to identify distinct patterns in outcomes across diverse cancer types and to implement systematic neurocognitive screening tools. This review aims to operationalize *processing efficiency* skill impairment—or inefficient neural processing as measured by working memory and processing speed abilities—as a worthwhile avenue for continued study within the context of childhood cancer.

**Methods:**

A comprehensive literature review was conducted to examine the existing research on cognitive late effects and biopsychosocial risk factors in order to conceptualize processing efficiency skill trends in childhood cancer survivors.

**Results:**

While a frequently reported pattern of neurobiological (white matter) and cognitive (working memory and processing speed) disruption is consistent with processing efficiency skill impairment, these weaknesses have not yet been fully operationalized in this population. We offer a theoretical model that highlights the impacts of a host of biological and environmental factors on the underlying neurobiological substrates of cancer survivors that precede and may even predict long‐term cognitive outcomes and functional abilities following treatment.

**Conclusion:**

The unified construct of processing efficiency may be useful in assessing and communicating neurocognitive skills in both outcomes research and clinical practice. Deficits in processing efficiency may serve as a possible indicator of cognitive late effects and functional outcomes due to the unique relationship between processing efficiency skills and neurobiological disruption following cancer treatment. Continued research along these lines is crucial for advancing childhood cancer outcomes research and improving quality of life for survivors.

## Graphical abstract

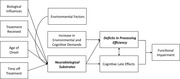

## 1 BACKGROUND

Considerable medical advancements in the treatment of childhood cancers have led to increased rates of survivorship despite increased incidence over time (Steliarova‐Foucher et al., 2017), with recent estimates indicating that about 84% of children with cancer now survive to live 5 years or more past diagnosis (American Cancer Society, 2020). With a significant majority of pediatric cancer patients now surviving and aging into adulthood, increasing efforts have been directed to improving quality of life by studying outcomes and identifying patterns of neurocognitive and psychological sequalae following treatment. Although research is steadily emerging, studies investigating long‐term outcomes have not kept pace with the significant treatment advancements in chemotherapy, radiation, and surgical interventions that have compelled increased rates in survivorship. The existing literature indicates that childhood cancer and cancer‐related treatments may disrupt brain development and maturation, placing survivors at risk for long‐term problems with cognitive functions (Gross‐King et al., 2008; Hardy et al., 2018; Jones & Pattwell, 2019; Krull et al., 2018; Marusak et al., 2018; Mulhern & Palmer, 2003; Perry & Schmidt, 2006). Difficulties with “thinking, learning, and remembering” that emerge following cancer treatment are often referred to as *cognitive late effects*, with impacts that may not be evident immediately and sometimes do not emerge until years after treatment (Mulhern & Palmer, 2003).

**FIGURE 1:**
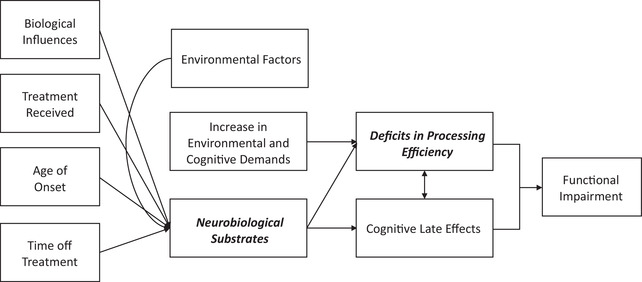
Conceptual framework: factors impacting cognitive late effects and functional abilities observed in pediatric cancer survivors. *Note*: This novel conceptual framework summarizes factors impacting the documented cognitive late effects in pediatric cancer survivors. Risk factors for neurobiological disruption (biological influences, treatment received, age of onset, time off treatment—see Sections 1.1 and 2.1) coupled with external factors and increase in demands lead to the emergence of cognitive late effects (see Section 1.2) as survivors age. Processing efficiency impairments (see Sections 2, 3, and 3) are one of the most consistently reported cognitive late effects in survivors and may serve as a possible indicator of functional impairment (see Section 6) in this population

The specific patterns of neurocognitive performance in pediatric cancer survivors remain to be fully elucidated, including potential risk factors for cognitive deficits, screening tools for assessing neurocognitive outcomes, and the translational impacts of these deficits on long‐term outcomes and functional independence. Cognitive late effects research to date has offered substantive evidence of a distinct pattern of inefficient neural processing in pediatric cancer survivors; however, this pattern has not yet been optimally conceptualized or operationalized for outcomes research or clinical application. Throughout this narrative review, we present support for a novel conceptual framework that relates a distinct cognitive construct—*processing efficiency*—with risk and environmental factors, neurobiology, and functional outcomes in pediatric cancer survivors (Figure 1).

### 1.1 Risk factors

It is currently appreciated that a combination of biological and environmental influences, including age of onset, cancer type and location, treatment approach, sex, genetic differences, and time off treatment, are all associated with cognitive and psychological outcomes following cancer treatment (Jones & Pattwell, 2019; Krull et al., 2018; Moore, 2005). The interplay of these risk factors for cognitive late effects is complex, and emerging research continually indicates that further study is warranted. For example, female sex was long believed to be a consistent significant risk factor for cognitive late effects (Mulhern & Palmer, 2003; Waber et al., 1990), which could be at least partly related to structural and functional brain differences in white matter pathways between sexes (Kanaan et al., 2012). However, more recently, research exploring sex differences has been highly variable, with female sex presenting as a risk factor in certain populations (Gandy et al., 2022; van der Plas et al., 2021), males and females sometimes demonstrating similar cognitive outcomes (Oswald & Bo, 2020), and even females’ neurocognitive skills improving significantly over time compared to males (Bledsoe et al., 2019). Further, sociodemographic variables, including ethnicity, income, and parental education, are related to outcomes for survival and cognitive late effects but remain largely understudied (Ahles & Root, 2018; Bhatia, 2004; Torres et al., 2021).

The additive pathways for neurobiological disruption in cancer patients include both the underlying disease pathology and the indirect, compounding effects of treatment. Pre‐treatment cancer‐related factors that influence cognitive outcomes include multimodal cytokine upregulation that promotes oxidative stress and peripheral inflammation, hormonal changes and endocrine dysfunction, and stress‐related biological changes to that lead to hypothalamic‐pituitary‐adrenal (HPA) axis disruption (Merriman et al., 2013). Experiencing childhood cancer has also been conceptualized as a form of early life adversity that negatively impacts long‐term psychological outcomes via disruptions to neural development above and beyond other chronic medical conditions or injuries (Marusak et al., 2018). The interaction of these factors with effects of neurotoxic treatments ultimately increases susceptibility for cognitive late effects following treatment.

Survivors of childhood brain tumors and leukemia are at especially high risk for long‐term cognitive effects due to the neurological impacts of cancer treatments on the developing central nervous system (CNS) (Castellino et al., 2014; Krull et al., 2018). For example, attention and processing speed skills are impacted following brain tumor resection (Steinlin et al., 2003), and complications related to resection (e.g., hydrocephalus, shunt placement, and infection) may result in injury to healthy brain tissue that impacts global cognitive functioning posttreatment (Chapman et al., 1995; Rijnen et al., 2019). Cranial radiation therapy (CRT) exposure and dosage is associated with a slew of neuroanatomical effects, including decreased white matter volume and connectivity, decreased cerebral perfusion (Mabbott et al., 2006), slower electroencephalogram (EEG) frequencies (Moore et al., 1992), and poorer performance on neuropsychological measures (Merchant et al., 2014; Mulhern & Palmer, 2003) that also correlate with differences in brain function and activation compared to healthy controls (Mabbott et al., 2006; Robinson et al., 2014). Non‐CNS cancer survivors also demonstrate clinically significant cognitive late effects due to the neurotoxic effects of chemotherapies alone (Anderson & Kunin‐Batson, 2009; Copeland et al., 1996; Mohrmann et al., 2015), with certain chemotherapies used in high doses or administered intrathecally increasing risk of long‐term cognitive dysfunction (Sleurs et al., 2016). Across both CNS and non‐CNS cancer types, these cognitive disruptions are associated with diffuse alterations in brain structure and function following treatment (Kesler et al., 2021).

Informed by developmental cognitive neuroscience, age of cancer onset has emerged as a consistent risk factor for cognitive late effects; the younger a child is at the time of cancer diagnosis, the greater their risk for intellectual, cognitive, and psychological disruption (Chapman et al., 1995; Jones & Pattwell, 2019; Moore, 2005). Efficient brain processes are dependent on intact white matter pathways, and disruptions to white matter integrity during childhood negatively impact later development of interactive processes across connected brain regions. Through a neurodevelopmental lens, younger brains are more susceptible to chemical imbalances that may disrupt new and developing myelination (Marusak et al., 2018), and the frontal lobe is most sensitive to effects of radiation primarily because this brain region continues to myelinate through young adulthood (Ullén, 2009). Within the cancer outcomes research, detrimental cognitive effects become increasingly evident over time, and longer time since diagnosis and treatment are related to worse cognitive outcomes (Krull et al., 2018). Altogether, the current evidence suggests the interaction of biopsychosocial factors and the widespread disruption of white matter pathways following cancer treatment contribute to the cognitive phenotype reflective of inefficient neural processing observed among childhood cancer survivors.

### 1.2 Trends in cognitive late effects

To date, the wide‐ranging documented cognitive late effects across both CNS and non‐CNS cancer types include deficits in executive functioning, attention, working memory, processing speed, memory, visual‐spatial, and motor skills, as well as decline in global intellectual abilities and general ability to learn (Anderson & Kunin‐Batson, 2009; Askins et al., 2015; Castellino et al., 2014; Gross‐King et al., 2008; Hardy et al., 2018; Hardy et al., 2018; Jones & Pattwell, 2019; Kadan‐Lottick et al., 2010; Kesler et al., 2021; Krull et al., 2018; Marusak et al., 2018; Mulhern & Palmer, 2003; Patel et al., 2016; Robinson et al., 2010; Winter et al., 2014), with some estimates suggesting that up to 60% of pediatric cancer survivors demonstrate neurocognitive difficulties in at least one of these domains (American Cancer Society, 2003; Jacola et al., 2021). While some survivors do not demonstrate impairments over time, others exhibit a host of skill deficits that warrant services and intervention and may impact long‐term quality of life (Butler & Copeland, 2006; Mitby et al., 2003). Those who demonstrate cognitive late effects are not necessarily declining or regressing in cognitive performance; instead, they demonstrate difficulty continuing to acquire age‐appropriate neurocognitive skills at the same rate as their peers, with a gap that continues to widen over time (Palmer et al., 2001). This pattern informs the need for close monitoring of neurocognitive skills following treatment in order to detect possible neurocognitive disruption and provide timely intervention. Unfortunately, the wide variability in outcomes has presented a challenge for efficient monitoring of this population.

The divergence in assessment tools selected by clinicians and researchers may explain some of the notable variability in outcomes beyond individual differences alone. Specifically, the inconsistent use of the term “executive functioning” in the cancer research literature is striking. Executive functioning is an umbrella term that encompasses an assortment of higher‐order skills needed for cognitive control and self‐regulation. Included under the umbrella of executive functioning are cognitive skills that assist with accomplishing a goal, such as modulation and regulation of attention, working memory, cognitive flexibility, inhibition, planning and organization, and self‐monitoring. While executive functioning is often cited as an area of deficit in pediatric cancer survivors (Copeland et al., 1996; Askins et al., 2015; Askins & Moore, 2008; Balsamo et al., 2019; Brinkman et al., 2012; Caron et al., 2009), a specific profile of skill deficits has yet to be determined because the actual executive functioning skills measured across sites and samples vary widely. For example, impairment in cognitive flexibility (Brinkman et al., 2012), organization (Caron et al., 2009), and abstract reasoning (Hollen et al., 2013) are all cited as broader weaknesses in “executive functioning” in this population, but each of these studies report a different pattern of performance across these higher‐order cognitive skills. The most consistent evidence for executive functioning deficits among pediatric cancer survivors appears to be from parent‐ or self‐ report ratings rather than lab‐based measures (Alderson & Mullins, 2011; Harman et al., 2019; Patel et al., 2013), and these studies typically report primary deficits in working memory skills that translate to broader difficulties with activities of daily living and functional independence.

Despite wide variability in cognitive outcomes, the most frequently reported deficits are across the domains of attention, processing speed, and working memory skills (Askins & Moore, 2008; Williams & Cole, 2021; Siegwart et al., 2020). Even early research investigating patterns of neurocognitive impairment in pediatric cancer survivors identified measures of working memory and processing speed as unique predictors that accounted for up to 45% of the variance in declining IQ scores among children with ALL treated with cranial radiation therapy (Schatz et al., 2000). Impairment in speed of information processing is perhaps the most consistent findings in this line of research (Krull et al., 2018), and widely observed deficits in short‐term memory and working memory negatively impact the ability to encode and retain new information. Weaknesses in these skill domains are observed across pediatric cancer survivors with diverse cancer presentation (i.e., CNS and non‐CNS cancer types) and cancer treatment history.

For instance, retrospective research on 20 adult survivors of childhood medulloblastoma suggests that cranial radiation is associated with significant effects on speeded processing and progressive decline in working memory skills long‐term that resemble the course of subcortical neurogenerative disease (Edelstein et al., 2011). A study utilizing principal component analysis to examine the neuropsychological performance of 82 pediatric brain tumor survivors revealed slowed processing as the primary impairment and underlying construct of neurocognitive deficits in survivors compared to sibling controls (de Ruiter et al., 2017). A meta‐analysis evaluating 509 ALL survivors at least 2 years off chemotherapy‐only treatment revealed significant impairment in the domains of working memory, information processing speed, and fine motor control compared to controls (Iyer et al., 2015). Another study investigating 23 survivors of childhood ALL treated with chemotherapy only revealed significant deficits in speed of information processing, with intact performance on measures of attention and accuracy across tasks compared to age‐ and sex‐matched controls (Mennes et al., 2005). Self‐report questionnaires assessing perceived neurocognitive functioning revealed task efficiency (which can be considered a form of processing speed) to be the greatest cognitive complaint in adult survivors of non‐CNS childhood cancer (Kadan‐Lottick et al., 2010), and survivors of pediatric CNS tumors reported working memory deficits to be the greatest self‐reported impairment impacting daily functioning (Brinkman et al., 2016).

Taken together, this body of evidence suggests a pattern of pervasive core deficits in *processing speed* and *working memory* skills among childhood cancer survivors with diverse cancer and treatment backgrounds. This pattern of skill deficit warrants a targeted investigation of processing efficiency skills across a mixed sample of pediatric cancer survivors. It is feasible that patterns of processing efficiency impairment may be related to other risk factors for cognitive late effects, may underlie broad learning and cognitive difficulties, and could perhaps act as a sensitive predictor for other long‐term cognitive effects.

### 1.3 Processing efficiency

Here, *processing efficiency* skills are presented as a form of cognitive proficiency measured by the brain's ability to process information quickly (processing speed) and hold information in mind for short periods of time (working memory). Skillful processing efficiency enables one to think and learn effectively, as cognitive resources in the mental workspace can be “freed up” to address more challenging cognitive tasks as they arise. Processing efficiency skills can be considered a distinct construct separate from the umbrella classification of executive functioning skills (Frischkorn et al., 2019). While executive functioning refers to broad higher‐order cognitive skills with variable patterns of skillsets, processing efficiency hones specifically on capacity for efficient neural information processing. Impairments in the subcomponents of processing efficiency skills (working memory and processing speed) are reported widely across studies of childhood cancer survivors; this is another qualitative factor that sets this skill apart from disparate reports of executive functioning skills in this population. Further, deficits in processing efficiency skills are regularly observed alongside white matter disruption, which presents the potential for this construct to be a sensitive indicator for neurobiological disturbance following cancer treatment.

### 1.4 Current review

A consistent theme across reviews of neurocognitive outcomes in childhood cancer survivors is a call to action; there remains a great need for empirical research that sheds light on the biological and developmental mechanisms underlying cognitive late effects in this population, with hopes for improving outcomes through risk‐identification and targeted interventions. Along these lines, a frequently reported but poorly defined trait in pediatric cancer survivors across the lifespan is impaired processing speed and working memory abilities—or, as we conceptualize here— *processing efficiency* skills. The purpose of this review is to define and operationalize processing efficiency skills within the context of childhood cancer survivors in order to inform its potential research and clinical utility. Specifically, the standardized use of this unified construct could support communication of neurocognitive outcomes across researchers and clinicians. Perhaps more ambitiously, processing efficiency may even emerge as an essential construct in developing a formal screening tool for monitoring cognitive outcomes. Investigations of cognitive screening tools in clinic settings are mixed, with some demonstrating poor sensitivity for detecting difficulties that may warrant full neuropsychological assessment and others requiring highly trained neuropsychologists to administer nuanced measures, indicating that there is a critical need for optimizing an accessible screening tool with good predictive value for cognitive late effects (Balsamo et al., 2019; Embry et al., 2012).

## 2 DEVELOPING A CONSENSUS DEFINITION FOR PROCESSING EFFICIENCY

Processing efficiency has been broadly characterized as the “optimal use of mental resources” that impacts ability to learn and coordinate goal‐directed behavior (Hoffman et al., 2012). Although most cognitive abilities are interrelated, skillful processing efficiency can uniquely promote learning and problem solving by “freeing up” cognitive resources for higher‐level cognitive processing (Weiss et al., 2016). Processing efficiency deficits are widely described across medical, neurodevelopmental, and neuropsychiatric conditions. To further describe the subcomponents of processing efficiency skills, processing speed is typically measured as the speed of cognitive processing via reaction times to stimuli or accurate completion of menial tasks within a certain time window, and working memory refers to the cognitive capacity to hold information in mind for a short period of time while also manipulating that information in one's mind in order to complete a task (Cowan, 2014). Modern Wechsler intelligence scales include the Cognitive Proficiency Index (CPI), an optional composite score that summarizes performance across measures of both processing speed and working memory (Wechsler, 2008; Wechsler, 2014). This index provides additional empirical support for our current operationalization of processing efficiency.

While processing efficiency is a distinct cognitive construct, it is also associated with other cognitive skills, including general intellectual ability (Weiss et al., 2016; Coyle, 2017; Scheuffgen et al., 2000), fluid reasoning (Demetriou et al., 2002), executive functioning (McCabe et al., 2010; Mulder et al., 2011), and general memory (Ramachandran, 2012). These relationships are difficult to extricate, but it is possible that processing efficiency impairment may precede, mediate, or even predict skill deficits across other domains in populations with neurodevelopmental differences. For example, outcomes research investigating cognitive profiles of children born premature suggests that broad executive dysfunction may be mediated by underlying disruption in processing speed (Mulder et al., 2011). Processing efficiency deficits can be pervasive and have the potential to negatively affect learning, social development, academic performance, and future vocational attainment (Giofrè & Cornoldi, 2015; Mayes & Calhoun, 2007; Phillips et al., 2007). For instance, children with impairments in processing efficiency may appear inattentive because they take longer to process stimuli, demonstrate difficulties following directions due to difficulties with recall or delayed processing, and struggle socially with difficulties engaging in reciprocal conversation that requires timely processing of input and continuous maintenance and refreshing of information in the mental workspace.

### 2.1 Neuroanatomy and brain function related to processing efficiency

Neural processing is both cognitively and computationally taxing, and thus requires efficiency of neural resources for optimal functioning achieved through a balance of functional specialization and functional integration (Battista et al., 2018; Tononi et al., 1998). This balance begins early in brain development, with brain regions becoming anatomically segregated across the cortex and connections forming across these regions through integration (Friston, 2011). Although higher‐order cognitive skills have historically been associated with functioning of the prefrontal and frontal cortex, it is increasing understood that a distributed network of brain regions including frontal, posterior, and parietal regions are recruited for efficient cognitive processing (Alvarez & Emory, 2006; Collette et al., 2006).

The link between aggressive cancer treatment and progressive white matter disruption appears to play a unique and critical role in neurocognitive outcomes (Askins & Moore, 2008). In addition to the white matter disruptions described earlier in patients who received CRT, high‐dose chemotherapies are also associated with damage to white matter integrity and leukoencephalopathy course (Perry & Schmidt, 2006; Reddick et al., 2005), and radiation and chemotherapy together predict lower white matter integrity in adulthood that is associated with lower intellectual functioning (King et al., 2015; Mulhern et al., 1999). Additionally, treatment‐related damage to neural progenitor cells (responsible for repairing damaged neurons in the hippocampus) and neuroglial cells (responsible for cell myelination in the CNS) are decreased following chemotherapy treatment and are uniquely associated with changes in processing speed and memory performance (Merriman et al., 2013). Disruptions to vasculature can also impact the availability of metabolic resources in the brain and limit effective cognitive processing, with possibilities for long‐term effects if these concerns lead to hypoxic episodes.

## 3 PROCESSING EFFICIENCY IN PEDIATRIC CANCER SURVIVORS

Pediatric cancer survivors who present for neuropsychological evaluation often present with a cognitive phenotype reflective of inefficient neural processing. Singular aspects of processing efficiency skills, including processing speed deficits and disruptions to working memory, are well documented across investigations of neurocognitive outcomes, but are often aggregated with other cognitive findings. There are several developmental models that propose aspects of processing efficiency may serve as mediators for cognitive late effects (Schatz et al., 2000; Palmer, 2008; Reddick et al., 2003; Wolfe et al., 2012; King et al., 2019). Processing speed and working memory are also often described separately from executive functions, suggesting that there is initial consensus that these cognitive skills are nuanced from general higher‐order skills. The conceptualization of processing efficiency skills among pediatric cancer survivors appears to lag behind the research of other neuropathological disorders that share similar neurobiological disruptions.

## 4 PROCESSING EFFICIENCY TRENDS ACROSS OTHER CHILDHOOD CONDITIONS

Impairments in processing efficiency have been described across other well‐researched childhood conditions with underlying neuropathology. These findings offer promising perspectives for evaluating and communicating cognitive outcomes within cancer populations. For example, within the anxiety literature, the Processing Efficiency Theory (PET) posits that anxiety and worry significantly disturb task performance; processing efficiency skills decline in anxious states due to the investment of additional cognitive resources to maintain accuracy, with both working memory and processing speed uniquely contributing to this effect (Eysenck & Calvo, 1992). Verbal working memory skills in children act as a significant mediator of the relationship between trait anxiety and academic performance (Owens et al., 2008). It is notable that the working memory weaknesses among pediatric cancer survivors are observed on both laboratory‐based and report‐based measures, suggesting an underlying deficit that may translate to broader academic challenges similar to impacts of mood disturbance explained by the PET.

The behavioral presentations of some neurodevelopmental disorders are theorized to manifest through inefficient neural connections and communication between brain regions (Castellanos & Aoki, 2016; Müller, 2007; Vasa et al., 2016). Differential performance on the Cognitive Proficiency Index on the WISC‐V among special groups suggests that individuals with ASD and ADHD demonstrate their weakest performance on measures of processing speed and working memory (Weiss et al., 2016). Survivors of childhood ALL and brain cancer referred for comprehensive neuropsychological evaluation are often diagnosed with secondary ADHD that negatively impacts functional independence and academic achievement (Reeves et al., 2007; Willard et al., 2013; Peterson et al., 2021). Given the notable overlap in presentation, previous developmental models have been proposed for considering cancer outcomes through the lens of ADHD. However, it is increasingly appreciated that the neurocognitive disruptions in cancer survivorship are distinct from those observed in developmental and secondary ADHD (Alderson & Mullins, 2011). A conceptual framework for cognitive late effects warrants additional consideration beyond what can be extracted from neurodevelopmental research alone.

Perhaps the most translational comparison for processing efficiency outcomes of survivors of pediatric cancer are among children who experience traumatic brain injury (TBI). In fact, the effects of aggressive cancer treatments can be conceptualized as an acquired brain injury due the significant impacts on cerebral white matter and vasculature (Corti et al., 2019). Deficits in information processing observed in TBI are secondary to the effects of diffuse white matter injury, with studies reporting decreased speeded processing and verbal fluency as well as reduced white matter volume and integrity in the corpus callosum (Weiss et al., 2016; Mathias et al., 2004; Wu et al., 2010). A popular computerized screening tool for sport‐related concussion (mild TBI) assesses skills across processing speed and working memory to determine return to baseline cognitive functioning before initiating a return to play protocol (Allen & Gfeller, 2011). The achievements along this line of research provide a hopeful bridge for potential adaptation to outcomes research within pediatric cancer survivorship.

## 5 PROCESSING EFFICIENCY AND COGNITIVE AGING

The processing‐speed theory of cognitive aging is a well‐established model for conceptualizing age‐related cognitive decline (Salthouse, 1996). This model attributes general cognitive difficulties observed in healthy aging to primary deficits in speed of information processing. A neuroanatomical explanation for cognitive slowing is the gradual disruptions to white matter integrity displayed over time—often referred to as the white matter degeneration hypothesis (Madden et al., 2009; Salami et al., 2012). Within neurodegenerative disorders, multiple sclerosis (MS) is a demyelinating disease that is often characterized by varying levels of cognitive impairment that may be accounted primarily by slowed speed of information processing (Drew et al., 2009). Screening tools that include measures of processing speed have been developed for assessing risk for dementia in Parkinson's disease and have also been associated with decreased functional connectivity (Jalakas et al., 2019).

These findings are relevant for the study of childhood cancer survivors given mounting evidence of accelerated cognitive aging in this population (Guida et al., 2019). Increasing efforts are directed towards preventing or treating the potential adverse neurologic aging effects of cancer and cancer treatment (Guida et al., 2021). A staggering 10–20% of survivors of childhood cancer demonstrate early onset frailty, or reduced physiologic reserve measured by slowness, weakness, low skeletal muscle mass, and exhaustion that increase risk for chronic disease and disability (Henderson et al., 2014; Smitherman et al., 2020; Williams et al., 2021). Cytotoxic chemotherapy treatment is associated with alterations to DNA that impact brain aging and changes in cognitive functioning (Kovalchuk & Kolb, 2017). It is possible that observed impairments in both physiological and cognitive skills in cancer survivorship are related to underlying neurobiological disruptions that are shared with other neurodegenerative disease. These findings collectively support the utility of routine monitoring of processing efficiency skills in cancer survivors across the lifespan.

## 6 PROPOSAL FOR OPERATIONALIZATION OF PROCESSING EFFICIENCY IN PEDIATRIC CANCER SURVIVORS

Processing efficiency is not a novel construct; it is well conceptualized across other conditions with underlying neuropathology and has been utilized within both research and clinical applications. Here, we highlight how this construct has not yet been integrated in the pediatric cancer outcomes literature, despite frequently reported impairments in both processing speed and working memory coupled with white matter disruption across survivors of diverse cancer types and treatment backgrounds. We propose a universal and unifying framework for researchers and clinicians to assess and communicate this pattern of inefficient neural processing. Applications of this construct in this population would unify researchers and clinicians in their use of language and assessment tools, which may eventually enable comparisons across studies of diverse cancer types. Although evidence suggests processing efficiency impairment is a shared weakness, we do anticipate differential performance on processing efficiency measures between cancer types. A targeted study of this shared cognitive late effect across cancer types may allow for nuanced examination of complex risk factors and underlying mechanisms. Identifying these patterns and associations with factors beyond CNS status is worthwhile. Lastly, we propose that processing efficiency skill assessment will be an essential component to any first‐level screener that may indicate whether more comprehensive evaluation is needed.

With increased rates of childhood cancer survivorship and advances in understanding of neurotoxic treatment effects, the Children's Oncology Group has recommended regular neuropsychological evaluation to monitor neurocognitive skills over time (Children's Oncology Group, 2018). Specifically, formal neuropsychological evaluation is clearly recommended for certain therapeutic exposures (i.e., head/brain radiation, neurosurgery, methotrexate, and cytarabine) and is further recommended “as indicated” for any other cancer experience across childhood, adolescence, or young adulthood. Comprehensive neuropsychological evaluation is often warranted to fully elucidate neurocognitive profiles and inform treatment planning. However, routine evaluations are often challenging to carry out in practice due to factors such as cost and access to services and specialty providers (Embry et al., 2012). There is great need for an effective and accessible screening tool for monitoring cognitive late effects following a prevention‐based model (Hardy et al., 2017). Outcomes of a brief screening tool could inform treatment planning and effectively triage patients with referral needs for comprehensive neuropsychological evaluation.

Evidence to date suggests that a standardized assessment of processing efficiency skills may be a prime candidate for a brief neurocognitive screening battery. Disruption to processing efficiency skills may be assessed through measures that tap into underlying processing speed and working memory abilities that may be impaired due to neurotoxic effects of cancer and cancer‐related treatment. A processing efficiency screening battery could be assembled with standardized psychometric tools that are widely available, and may even be utilized by professionals who may not be specifically trained in neuropsychology. Although this has not yet been a focus of targeted study, it is anticipated that a portion of pediatric cancer survivors will demonstrate deficits in processing efficiency skills that may be related to other cognitive skills following cancer treatment. Although screening tools within this population have not yet been optimized, there is one study with aspects worth discussion in this review.

Krull et al. (2008) evaluated the predictive and discriminative validity of a brief screening tool to evaluate neurocognitive outcomes in pediatric cancer survivors. This screening measure, coined “DIVERGT,” focused on skills across the domains of auditory working memory (Digit Span), executive functioning (Verbal Fluency), and speeded processing on tasks involving fine motor control (Grooved Pegboard) and visuomotor sequencing (Trails). Outcomes of this screening measure were tested across 240 survivors of diverse pediatric cancer types (leukemia, lymphoma, CNS tumors, and non‐CNS tumors) and revealed almost half of the sample (47.9%) demonstrated some level of impairment on the DIVERGT screener (i.e., one standard score < 70 or two standard scores < 80). Among a smaller sample of 52 patients reevaluated for comprehensive neuropsychological evaluation, results indicated that this screening tool successfully predicted global intellectual functioning and academic skills in reading and mathematics. Assessment of test–retest reliability, sensitivity, and specificity were all robust for this screening tool, which was also a significantly stronger predictor of outcomes than parent ratings of behavioral, emotional, academic, and cognitive symptoms on the Child Symptom Inventory (CSI). Overall, this study provided compelling evidence for the predictive utility of a brief screening tool to assess aspects of processing speed, working memory, and verbal fluency that may inform need for comprehensive evaluation.

Although not specifically operationalized in Krull and colleagues’ paper, the measures used are all conceptually consistent with the construct of *processing efficiency*. These findings provide additional empirical support for our framework and clearly demonstrate that processing efficiency skills will be essential to any effective screening tool utilized in this population. While these findings are compelling, observed deficits have yet to be conceptualized explicitly under the unified construct of processing efficiency or the underlying neurobiological disruption related to cancer and cancer‐related treatment. The translational impacts of these deficits have also not yet been investigated beyond prediction of global intellect and academic performance in reading and math.

### 6.1 Conceptual model: The role of processing efficiency in cognitive late effects

Known risk factors for cognitive late effects in pediatric cancer survivors include biological influences (age of onset, sex, genetics etc.), treatment approach (i.e., neurosurgery, cranial radiation, chemotherapy, multimodal), and time off treatment. Over time, disruptions to developing neurobiological substrates (i.e., white matter integrity) coupled with external factors (e.g., socioeconomic status, access to services, etc.) and general increase in cognitive and environmental demands as survivors age ultimately lead to the presentation of cognitive late effects. These long‐term cognitive deficits are observed in a significant portion of childhood cancer survivors (estimates up to 60%) and lead to functional impairment across settings, including academic, vocational, social, and general adaptive skills (American Cancer Society, 2003). Processing efficiency impairments emerge as one of the most consistent cognitive late effects in both CNS and non‐CNS cancers. Due to the unique relationship between processing efficiency skills and neurobiological disruption following cancer treatment, deficits in processing efficiency could serve as an indicator or possible predictor of cognitive late effects and functional outcomes; however, this remains to be studied and considered in the context of complex biopsychosocial factors. Figure 1 offers a framework that builds from existing conceptual models and summarizes these factors impacting cognitive late effects observed in pediatric cancer survivors.

## 7 CLINICAL IMPLICATIONS, LIMITATIONS, AND FUTURE DIRECTIONS

This review outlines the potential value of assessing and communicating processing efficiency skills among childhood cancer survivors. While the evidence is compelling, additional empirical support is required before the translational impacts of this theoretical framework can be fully understood and applied. Future directions in this line of research should first focus on characterizing a diverse clinical sample through the lens of this framework. It is anticipated that children presenting for comprehensive neuropsychological evaluation through standard clinical care following cancer treatment will demonstrate general processing efficiency deficits. As discussed here, these processing efficiency deficits can be identified through performance across standardized measures of processing speed, working memory, and semantic verbal fluency that are predicted to be below age‐based expectations. Factors that should be considered in follow‐up studies include investigating processing efficiency in both CNS and non‐CNS cancer survivors to determine whether there are differential profiles of processing efficiency among cancer types. As CNS‐directed therapies are related to increased rates of subsequent disruption to brain development and maturation, it is anticipated that processing efficiency deficits will be especially sensitive to outcomes of children receiving these neurotoxic treatments. Processing efficiency deficits among pediatric cancer survivors could also be investigated for their predictive utility for detecting impairment across other cognitive domains (e.g., executive functioning, memory, language processing, visual‐spatial skills, etc.), as intact processing efficiency skills are believed to “free up” cognitive reserve for higher‐order tasks. It is unlikely that deficits in processing efficiency directly cause these other cognitive deficits; rather, processing efficiency may act as a tool for detecting early disruption in neurocognitive functioning that may later fully manifest as these downstream cognitive late effects.

Another clinically meaningful application of this work will be to explore the translational impacts of individual differences in processing efficiency deficits in pediatric cancer survivors. It is anticipated that as deficits in processing efficiency increase, there will be greater likelihood of impairments across activities of daily living and quality of life, ultimately driving need for additional services, clinical care, and follow‐up. Individual differences, such as demographic and environmental factors, are expected to drive some of the variability in processing efficiency skills following cancer treatment. Neuroimaging research specifically investigating the relationships between processing efficiency skills and white matter integrity and volume in survivors would be especially compelling. Longitudinal investigations of processing efficiency across time will yield crucial evidence for understanding the trajectories of disruptions to cognitive skills and possibly provide clues for the changes to underlying neurobiological substrates following cancer treatment.

Interventions may specifically target processing efficiency skills in this population. For example, a cognitive remediation program focused on targeted skill development in the domain of working memory yielded positive benefit that translated to improvements in parent‐reports of learning problems among a group of school‐aged ALL and brain tumor survivors (Hardy et al., 2013). Along those lines, it would be worthwhile for remediation and intervention studies to include processing efficiency as an outcome variable. There is a wealth of research documenting the neural plasticity of children and the potential for brain‐related changes, such as increased functional connectivity, through behavioral intervention (Murdaugh et al., 2015; Yuan et al., 2017). In fact, one study found that cognitive training focused on development of working memory and processing speed skills was associated with observable changes to white matter integrity, specifically in the anterior part of the corpus callosum (Lövdén et al., 2010). Outcomes of targeted cognitive remediation for information processing deficits in patients with a history of traumatic brain injury are noted to be related to increased recruitment of frontal and temporal regions and related functional connectivity (Ashley et al., 2012). It is increasingly plausible that processing efficiency may be a correlate of neural plasticity and could be used as a behavioral measure to indicate patterns of functional integration in the brain.

## 8 CONCLUSION

Research on cognitive late effects among survivors of pediatric cancer has gained recent and warranted momentum. Processing efficiency, an integrated and nuanced cognitive skill that has the potential to be an emerging hallmark sign of cognitive disruption in this population, remains largely understudied. Within this review, we have presented compelling theoretical evidence for the utility of processing efficiency assessment in pediatric cancer survivors. A novel conceptual framework for considering and describing processing efficiency skills among childhood cancer survivors has been proposed. It is well‐documented that biological influences, treatment approach, age of onset, and time off treatment are each uniquely associated with cognitive late effects, and that these risk factors are suspected to act through disruption to neurobiological substrates—primarily, white matter disruption. The framework offered here outlines the associations between neurobiological disruptions and the increase in environmental and cognitive demands that typically accompany cognitive late effects. Utilizing a standard approach for assessing and communicating processing efficiency skills across outcomes research and within routine clinical practice may allow for distinct patterns in neurocognitive outcomes to surface, with findings that may hold significant translational impacts for cognitive and adaptive outcomes. More ambitiously, outcomes of a processing efficiency screening measure may even serve as unique and sensitive tool for monitoring the emergence of cognitive late effects. Future directions of this line of research should take aim at providing empirical support for this characterization of processing efficiency impairment in childhood cancer populations while also considering associations with complex biopsychosocial factors known to impact survivorship outcomes.

## CONFLICT OF INTEREST

The authors of this paper have no conflicts of interest to declare, financial or otherwise. All contributors have read and approved this submission.

### PEER REVIEW

The peer review history for this article is available at https://publons.com/publon/10.1002/brb3.2809

## Data Availability

Data sharing not applicable to this article as no datasets were generated or analyzed during the current study.

## References

[brb32809-bib-0001] Ahles, T. A. , & Root, J. C. (2018). Cognitive effects of cancer and cancer treatments. Annual Review of Clinical Psychology, 14, 425–451. 10.1146/annurev-clinpsy-050817-084903 PMC9118140 29345974

[brb32809-bib-0002] Alderson, R. M. , & Mullins, L. L. (2011). Theoretical and clinical implications of using an ADHD framework to understand attention, concentration, and executive functioning deficits in pediatric cancer survivors. Pediatric Blood & Cancer, 57(1), 4–5. 21400656 10.1002/pbc.23061

[brb32809-bib-0003] Allen, B. J. , & Gfeller, J. D. (2011). The immediate post‐concussion assessment and cognitive testing battery and traditional neuropsychological measures: A construct and concurrent validity study. Brain Injury, 25(2), 179–191. 21219090 10.3109/02699052.2010.541897

[brb32809-bib-0004] Alvarez, J. A. , & Emory, E. (2006). Executive function and the frontal lobes: A meta‐analytic review. Neuropsychology Review, 16(1), 17–42. 16794878 10.1007/s11065-006-9002-x

[brb32809-bib-0005] American Cancer Society . (2020). Cancer facts fig 2020. Published online.

[brb32809-bib-0006] Anderson, F. S. , & Kunin‐Batson, A. S. (2009). Neurocognitive late effects of chemotherapy in children: The past 10 years of research on brain structure and function. Pediatric Blood & Cancer, 52(2), 159–164. 18680151 10.1002/pbc.21700

[brb32809-bib-0007] Ashley, M. J. , Ashley, J. , & Kreber, L. (2012). Remediation of information processing following traumatic brain injury: A community‐based rehabilitation approach. Neurorehabilitation, 31(1), 31–39. 22523011 10.3233/NRE-2012-0772

[brb32809-bib-0008] Askins, M. A. , Ann‐Yi, S. , & Moore, B. D. (2015). Neurocognitive late effects in children treated for cancer: Psychological impact, identification, and prevention and remediation. In G. A. Mucci , & L. R. Torno (Eds.), Handbook of long term care of the childhood cancer survivor (pp. 397–409). New York, NY, USA: Springer Science + Business Media.

[brb32809-bib-0009] Askins, M. A. , & Moore, B. D. (2008). Preventing neurocognitive late effects in childhood cancer survivors. Journal of Child Neurology, 23(10), 1160–1171. 18952582 10.1177/0883073808321065 PMC3674758

[brb32809-bib-0010] Balsamo, L. M. , Mitchell, H. ‐R. , Ross, W. , Metayer, C. , Hardy, K. K. , & Kadan‐Lottick, N. S. (2019). Monitoring neurocognitive functioning in childhood cancer survivors: Evaluation of Cogstate computerized assessment and the Behavior Rating Inventory of Executive Function (BRIEF). BMC Psychology , 7. 10.1186/s40359-019-0302-3 PMC6498488 31046815

[brb32809-bib-0011] Battista, C. , Evans, T. M. , Ngoon, T. J. , Chen, T. , Chen, L. , Kochalka, J. , & Menon, V. (2018). Mechanisms of interactive specialization and emergence of functional brain circuits supporting cognitive development in children. npj Science of Learning, 3(1), 1–11. 30631462 10.1038/s41539-017-0017-2 PMC6220196

[brb32809-bib-0012] Bhatia, S. (2004). Influence of race and socioeconomic status on outcome of children treated for childhood acute lymphoblastic leukemia. Current Opinion in Pediatrics, 16(1), 9–14. 14758108 10.1097/00008480-200402000-00004

[brb32809-bib-0013] Bledsoe, J. C. , Breiger, D. , Breiger, M. , Shonka, S. , Ermoian, R. P. , Ojemann, J. G. , Werny, D. M. , Leary, S. E. S. , & Geyer, J. R. (2019). Differential trajectories of neurocognitive functioning in females versus males following treatment for pediatric brain tumors. Neuro‐Oncology, 21(10), 1310–1318. 31123753 10.1093/neuonc/noz092 PMC6784260

[brb32809-bib-0014] Brinkman, T. M. , Krasin, M. J. , Liu, W. , Armstrong, G. T. , Ojha, R. P. , Sadighi, Z. S. , Gupta, P. , Kimberg, C. , Srivastava, D. , Merchant, T. E. , Gajjar, A. , Robison, L. L. , Hudson, M. M. , & Krull, K. R. (2016). Long‐term neurocognitive functioning and social attainment in adult survivors of pediatric CNS tumors: Results from the St. Jude lifetime cohort study. American Journal of Clinical Oncology, 34(12), 1358–1367. 10.1200/JCO.2015.62.2589 PMC4933131 26834063

[brb32809-bib-0015] Brinkman, T. M. , Reddick, W. E. , Luxton, J. , Glass, J. O. , Sabin, N. D. , Srivastava, D. K. , Robison, L. L. , Hudson, M. M. , & Krull, K. R. (2012). Cerebral white matter integrity and executive function in adult survivors of childhood medulloblastoma. Neuro‐Oncology, 14(4), iv25–iv36. 23095827 10.1093/neuonc/nos214 PMC3480251

[brb32809-bib-0016] Butler, R. W. , & Copeland, D. R. (2006). Interventions for cancer late effects and survivorship. In R. T. Brown (Ed.), Comprehensive handbook of childhood cancer and sickle cell disease: A biopsychosocial approach (pp. 297–312). New York, NY, USA: Oxford University Press.

[brb32809-bib-0017] Caron, J. E. , Krull, K. R. , Hockenberry, M. , Jain, N. , Kaemingk, K. , & Moore, I. M. (2009). Oxidative stress and executive function in children receiving chemotherapy for acute lymphoblastic leukemia. Pediatric Blood & Cancer, 53(4), 551–556. 19499584 10.1002/pbc.22128 PMC3928629

[brb32809-bib-0018] Castellanos, F. X. , & Aoki, Y. (2016). Intrinsic functional connectivity in attention‐deficit/hyperactivity disorder: A science in development. Biological Psychiatry: Cognitive Neuroscience and Neuroimaging, 1(3), 253–261. 27713929 10.1016/j.bpsc.2016.03.004 PMC5047296

[brb32809-bib-0019] Castellino, S. M. , Ullrich, N. J. , Whelen, M. J. , & Lange, B. J. (2014). Developing interventions for cancer‐related cognitive dysfunction in childhood cancer survivors. The Journal of the National Cancer Institute (JNCI), 106(8),. 10.1093/jnci/dju186 PMC4155432 25080574

[brb32809-bib-0020] Chapman, C. A. , Waber, D. P. , Bernstein, J. H. , Pomeroy, S. L. , Lavally, B. , Sallan, S. E. , & Tarbell, N. (1995). Neurobehavioral and neurologic outcome in long‐term survivors of posterior fossa brain tumors: Role of age and perioperative factors. Journal of Child Neurology, 10(3), 209–212. 7642890 10.1177/088307389501000308

[brb32809-bib-0021] Children's Oncology Group . (2018). Long‐term follow‐up guidelines for survivors of childhood, adolescent, and young adult cancers, version 5.0. Published online. 10.1001/jamaoncol.2024.6812 PMC12188901 39976936

[brb32809-bib-0022] Collette, F. , Hogge, M. , & Salmon, E. (2006). Exploration of the neural substrates of executive functioning by functional neuroimaging. Published online. 23. 10.1016/j.neuroscience.2005.05.035 16324796

[brb32809-bib-0023] Copeland, D. R. , Moore, B. D. , Francis, D. J. , Jaffe, N. , & Culbert, S. J. (1996). Neuropsychologic effects of chemotherapy on children with cancer: A longitudinal study. American Journal of Clinical Oncology, 14(10), 2826–2835. 10.1200/JCO.1996.14.10.2826 8874345

[brb32809-bib-0024] Corti, C. , Oldrati, V. , Oprandi, M. C. , Ferrari, E. , Poggi, G. , Borgatti, R. , Urgesi, C. , & Bardoni, A. (2019). Remote technology‐based training programs for children with acquired brain injury: A systematic review and a meta‐analytic exploration. Behavioural Neurology, 2019, 1–31. 10.1155/2019/1346987 PMC6701292 31467613

[brb32809-bib-0025] Cowan, N. (2014). Working memory underpins cognitive development, learning, and education. Educational Psychology Review, 26(2), 197–223. 25346585 10.1007/s10648-013-9246-y PMC4207727

[brb32809-bib-0026] Coyle, T. (2017). A differential–developmental model (DDM): Mental speed, attention lapses, and general intelligence. Journal of Intelligence, 5(2), 25. 31162416 10.3390/jintelligence5020025 PMC6526483

[brb32809-bib-0027] Demetriou, A. , Christou, C. , Spanoudis, G. , & Platsidou, M. (2002). The development of mental processing: Efficiency, working memory, and thinking. Monographs of the Society for Research in Child Development, 67(1), 1–38. 12360826

[brb32809-bib-0028] De Ruiter, M. A. , Grootenhuis, M. A. , Van Mourik, R. , Maurice‐Stam, H. , Breteler, M. H. M. , Gidding, C. , Beek, L. R. , Granzen, B. , Van Vuurden, D. G. , Schouten‐Van Meeteren, A. Y. N. , & Oosterlaan, J. (2017). Timed performance weaknesses on computerized tasks in pediatric brain tumor survivors: A comparison with sibling controls. Child Neuropsychology, 23(2), 208–227. 26586548 10.1080/09297049.2015.1108395

[brb32809-bib-0029] Drew, M. A. , Starkey, N. J. , & Isler, R. B. (2009). Examining the link between information processing speed and executive functioning in multiple sclerosis. Archives of Clinical Neuropsychology, 24(1), 47–58. 19395356 10.1093/arclin/acp007

[brb32809-bib-0030] Edelstein, K. , Spiegler, B. J. , Fung, S. , Panzarella, T. , Mabbott, D. J. , Jewitt, N. , D'agostino, N. M. , Mason, W. P. , Bouffet, E. , Tabori, U. , Laperriere, N. , & Hodgson, D. C. (2011). Early aging in adult survivors of childhood medulloblastoma: Long‐term neurocognitive, functional, and physical outcomes. Neuro‐Oncology, 13(5), 536–545. 21367970 10.1093/neuonc/nor015 PMC3093335

[brb32809-bib-0031] Embry, L. , Annett, R. D. , Kunin‐Batson, A. , Patel, S. K. , Sands, S. , Reaman, G. , & Noll, R. B. (2012). Implementation of multi‐site neurocognitive assessments within a pediatric cooperative group: Can it be done? Pediatric Blood & Cancer, 59(3), 536–539. 22555997 10.1002/pbc.24139

[brb32809-bib-0032] Eysenck, M. W. , & Calvo, M. G. (1992). Anxiety and performance: The processing efficiency theory. Cognition and Emotion, 6(6), 409–434.

[brb32809-bib-0033] Frischkorn, G. T. , Schubert, A. ‐L. , & Hagemann, D. (2019). Processing speed, working memory, and executive functions: Independent or inter‐related predictors of general intelligence. Intelligence, 75, 95–110.

[brb32809-bib-0034] Friston, K. J. (2011). Functional and effective connectivity: A review. Brain Connect, 1(1), 13–36. 22432952 10.1089/brain.2011.0008

[brb32809-bib-0035] Gandy, K. , Scoggins, M. A. , Phillips, N. , Van Der Plas, E. , Fellah, S. , Jacola, L. M. , Pui, C. ‐H. , Hudson, M. M. , Reddick, W. E. , Sitaram, R. , & Krull, K. R. (2022). Sex‐based differences in functional brain activity during working memory in survivors of pediatric acute lymphoblastic leukemia. JNCI Cancer Spectrum, 6(2), pkac026. 35603857 10.1093/jncics/pkac026 PMC9041337

[brb32809-bib-0036] Giofrã¨, D. , & Cornoldi, C. (2015). The structure of intelligence in children with specific learning disabilities is different as compared to typically development children. Intelligence, 52, 36–43.

[brb32809-bib-0037] Gross‐King, M. , Booth‐Jones, M. , & Couluris, M. (2008). Neurocognitive impairment in children treated for cancer: How do we measure cognitive outcomes? Journal of Pediatric Oncology Nursing, 25(4), 227–232. 18559886 10.1177/1043454208321114

[brb32809-bib-0038] Guida, J. L. , Agurs‐Collins, T. , Ahles, T. A. , Campisi, J. , Dale, W. , Demark‐Wahnefried, W. , Dietrich, J. , Fuldner, R. , Gallicchio, L. , Green, P. A. , Hurria, A. , Janelsins, M. C. , Jhappan, C. , Kirkland, J. L. , Kohanski, R. , Longo, V. , Meydani, S. , Mohile, S. , Niedernhofer, L. J. , … Ness, K. K. (2021). Strategies to prevent or remediate cancer and treatment‐related aging. JNCI: Journal of the National Cancer Institute, 113(2), 112–122. 32348501 10.1093/jnci/djaa060 PMC7850536

[brb32809-bib-0039] Guida, J. L. , Ahles, T. A. , Belsky, D. , Campisi, J. , Cohen, H. J. , Degregori, J. , Fuldner, R. , Ferrucci, L. , Gallicchio, L. , Gavrilov, L. , Gavrilova, N. , Green, P. A. , Jhappan, C. , Kohanski, R. , Krull, K. , Mandelblatt, J. , Ness, K. K. , O'Mara, A. , Price, N. , … Hurria, A. (2019). Measuring aging and identifying aging phenotypes in cancer survivors. JNCI: Journal of the National Cancer Institute, 111(12), 1245–1254. 31321426 10.1093/jnci/djz136 PMC7962788

[brb32809-bib-0040] Hardy, K. K. , Olson, K. , Cox, S. M. , Kennedy, T. , & Walsh, K. S. (2017). Systematic review: A prevention‐based model of neuropsychological assessment for children with medical illness. Journal of Pediatric Psychology, 42(8), 815–822. 28369473 10.1093/jpepsy/jsx060 PMC7328686

[brb32809-bib-0041] Hardy, K. K. , Willard, V. W. , Allen, T. M. , & Bonner, M. J. (2013). Working memory training in survivors of pediatric cancer: A randomized pilot study. Psycho‐Oncology, 22(8), 1856–1865. 23203754 10.1002/pon.3222 PMC4131851

[brb32809-bib-0042] Hardy, S. J. , Krull, K. R. , Wefel, J. S. , & Janelsins, M. (2018). Cognitive changes in cancer survivors. American Society of Clinical Oncology Educational Book, 38, 795–806. 30231372 10.1200/EDBK_201179

[brb32809-bib-0043] Harman, J. L. , Molnar, A. E. , Cox, L. E. , Jurbergs, N. , Russell, K. M. , Wise, J. , & Willard, V. W. (2019). Parent‐reported executive functioning in young children treated for cancer. Child Neuropsychology, 25(4), 548–560. 30049262 10.1080/09297049.2018.1503647

[brb32809-bib-0044] Henderson, T. O. , Ness, K. K. , & Cohen, H. J. (2014). Accelerated aging among cancer survivors: From pediatrics to geriatrics. American Society of Clinical Oncology Educational Book, 34, e423–e430. 10.14694/EdBook_AM.2014.34.e423 24857133

[brb32809-bib-0045] M. Hewitt , S. L. Weiner , & J. V. Simone (Eds.). (2003). Childhood cancer survivorship: Improving care and quality of life. National Academies Press. 25057670

[brb32809-bib-0046] Hoffman, B. , Schraw, G. , & McCrudden, M. T. (2012). Cognitive efficiency. In N. M. Seel (Ed.), Encyclopedia of the sciences of learning (pp. 590–593). Springer US.

[brb32809-bib-0047] Hollen, P. J. , Tyc, V. L. , Shannon, S. V. , Donnangelo, S. F. , Hobbie, W. L. , Hudson, M. M. , O'Laughlen, M. C. , Smolkin, M. E. , & Petroni, G. R. (2013). Factors related to decision making and substance use in adolescent survivors of childhood cancer: A presenting clinical profile. Journal of Cancer Survivorship, 7(3), 500–510. 23712611 10.1007/s11764-013-0287-5

[brb32809-bib-0048] Ramachandran, V. S. (2012). Aging and cognition. In S. Horning , & H. P. Davis (Eds.), Encyclopedia of human behavior (pp. 44–52). Elsevier.

[brb32809-bib-0049] Iyer, N. S. , Balsamo, L. M. , Bracken, M. B. , & Kadan‐Lottick, N. S. (2015). Chemotherapy‐only treatment effects on long‐term neurocognitive functioning in childhood ALL survivors: A review and meta‐analysis. Blood, 126(3), 346–353. 26048910 10.1182/blood-2015-02-627414

[brb32809-bib-0050] Jacola, L. M. , Partanen, M. , Lemiere, J. , Hudson, M. M. , & Thomas, S. (2021). Assessment and monitoring of neurocognitive function in pediatric cancer. Journal of Clinical Oncology, 39(16), 1696–1704. 33886364 10.1200/JCO.20.02444 PMC8260911

[brb32809-bib-0051] Jalakas, M. , Palmqvist, S. , Hall, S. , Svã¤Rd, D. , Lindberg, O. , Pereira, J. B. , Van Westen, D. , & Hansson, O. (2019). A quick test of cognitive speed can predict development of dementia in Parkinson's disease. Science Reports, 9(1), 15417. 10.1038/s41598-019-51505-1 PMC6817840 31659172

[brb32809-bib-0052] Jones, R. M. , & Pattwell, S. S. (2019). Future considerations for pediatric cancer survivorship: Translational perspectives from developmental neuroscience. Developmental Cognitive Neuroscience, 38, 100657. 31158802 10.1016/j.dcn.2019.100657 PMC6697051

[brb32809-bib-0053] Kadan‐Lottick, N. S. , Zeltzer, L. K. , Liu, Q. i. , Yasui, Y. , Ellenberg, L. , Gioia, G. , Robison, L. L. , & Krull, K. R. (2010). Neurocognitive functioning in adult survivors of childhood non‐central nervous system cancers. Journal of the National Cancer Institute (JNCI), 102(12), 881–893. 20458059 10.1093/jnci/djq156 PMC2886093

[brb32809-bib-0054] Kanaan, R. A. , Allin, M. , Picchioni, M. , Barker, G. J. , Daly, E. , Shergill, S. S. , Woolley, J. , & Mcguire, P. K. (2012). Gender differences in white matter microstructure. *PLoS One*, e38272, 7(6). 10.1371/journal.pone.0038272 PMC3368921 22701619

[brb32809-bib-0055] Kesler, S. R. , Sleurs, C. , Mcdonald, B. C. , Deprez, S. , Van Der Plas, E. , & Nieman, B. J. (2021). Brain imaging in pediatric cancer survivors: Correlates of cognitive impairment. Journal of Clinical Oncology, 39(16), 1775–1785. 33886371 10.1200/JCO.20.02315 PMC8260924

[brb32809-bib-0056] King, T. Z. , Ailion, A. S. , Fox, M. E. , & Hufstetler, S. M. (2019). Neurodevelopmental model of long‐term outcomes of adult survivors of childhood brain tumors. Child Neuropsychology, 25(1), 1–21. 28956496 10.1080/09297049.2017.1380178

[brb32809-bib-0057] King, T. Z. , Wang, L. , & Mao, H. (2015). Disruption of white matter integrity in adult survivors of childhood brain tumors: Correlates with long‐term intellectual outcomes. PLoS One, 10(7), e0131744. 26147736 10.1371/journal.pone.0131744 PMC4492692

[brb32809-bib-0058] Kovalchuk, A. , & Kolb, B. (2017). Chemo brain: From discerning mechanisms to lifting the brain fog—An aging connection. Cell Cycle, 16(14), 1345–1349. 28657421 10.1080/15384101.2017.1334022 PMC5539816

[brb32809-bib-0059] Krull, K. R. , Hardy, K. K. , Kahalley, L. S. , Schuitema, I. , & Kesler, S. R. (2018). Neurocognitive outcomes and interventions in long‐term survivors of childhood cancer. Journal of Clinical Oncology, 36(21), 2181–2189. 29874137 10.1200/JCO.2017.76.4696 PMC6553837

[brb32809-bib-0060] Krull, K. R. , Okcu, M. F. , Potter, B. , Jain, N. , Dreyer, Z. , Kamdar, K. , & Brouwers, P. (2008). Screening for neurocognitive impairment in pediatric cancer long‐term survivors. Journal of Clinical Oncology, 26(25), 4138–4143. 18757327 10.1200/JCO.2008.16.8864

[brb32809-bib-0061] Lövdén, M. , Bodammer, N. C. , Kühn, S. , Kaufmann, J. , Schütze, H. , Tempelmann, C. , Heinze, H. ‐ J. , Düzel, E. , Schmiedek, F. , & Lindenberger, U. (2010). Experience‐dependent plasticity of white‐matter microstructure extends into old age. Neuropsychologia, 48(13), 3878–3883. 20816877 10.1016/j.neuropsychologia.2010.08.026

[brb32809-bib-0062] Mabbott, D. J. (2006). Diffusion tensor imaging of white matter after cranial radiation in children for medulloblastoma: Correlation with IQ. Neuro‐Oncology, 8(3), 244–252. 16723629 10.1215/15228517-2006-002 PMC1871945

[brb32809-bib-0063] Madden, D. J. , Bennett, I. J. , & Song, A. W. (2009). Cerebral white matter integrity and cognitive aging: Contributions from diffusion tensor imaging. Neuropsychology Review, 19, 415–435. 19705281 10.1007/s11065-009-9113-2 PMC2787975

[brb32809-bib-0064] Marusak, H. A. , Iadipaolo, A. S. , Harper, F. W. , Elrahal, F. , Taub, J. W. , Goldberg, E. , & Rabinak, C. A. (2018). Neurodevelopmental consequences of pediatric cancer and its treatment: Applying an early adversity framework to understanding cognitive, behavioral, and emotional outcomes. Neuropsychology Review, 28(2), 123–175. 29270773 10.1007/s11065-017-9365-1 PMC6639713

[brb32809-bib-0065] Mathias, J. L. , Bigler, E. D. , Jones, N. R. , Bowden, S. C. , Barrett‐Woodbridge, M. , Brown, G. C. , & Taylor, D. J. (2004). Neuropsychological and information processing performance and its relationship to white matter changes following moderate and severe traumatic brain injury: A preliminary study. Applied Neuropsychology, 11(3), 134–152. 15590348 10.1207/s15324826an1103_2

[brb32809-bib-0066] Mayes, S. D. , & Calhoun, S. L. (2007). Learning, attention, writing, and processing speed in typical children and children with ADHD, autism, anxiety, depression, and oppositional‐defiant disorder. Child Neuropsychology: A Journal on Normal and Abnormal Development in Childhood and Adolescence, 13(6), 469–493. 17852125 10.1080/09297040601112773

[brb32809-bib-0067] Mccabe, D. P. , Roediger, H. L. , Mcdaniel, M. A. , Balota, D. A. , & Hambrick, D. Z. (2010). The relationship between working memory capacity and executive functioning: Evidence for a common executive attention construct. Neuropsychology, 24(2), 222–243. 20230116 10.1037/a0017619 PMC2852635

[brb32809-bib-0068] Mennes, M. , Stiers, P. , Vandenbussche, E. , Vercruysse, G. , Uyttebroeck, A. , De Meyer, G. , & Van Cool, S. W (2005). Attention and information processing in survivors of childhood acute lymphoblastic leukemia treated with chemotherapy only. Pediatric Blood & Cancer, 44(5), 478–486. 15918215 10.1002/pbc.20147

[brb32809-bib-0069] Merchant, T. E. , Schreiber, J. E. , Wu, S. , Lukose, R. , Xiong, X. , & Gajjar, A. (2014). Critical combinations of radiation dose and volume predict intelligence quotient and academic achievement scores after craniospinal irradiation in children with medulloblastoma. International Journal of Radiation and Oncology in Biology and Physics, 90(3), 554–561. 10.1016/j.ijrobp.2014.06.058 PMC4703579 25160611

[brb32809-bib-0070] Merriman, J. D. , Von Ah, D. , Miaskowski, C. , & Aouizerat, B. E. (2013). Proposed mechanisms for cancer‐ and treatment‐related cognitive changes. Seminars in Oncology Nursing, 29(4), 260–269. 24183157 10.1016/j.soncn.2013.08.006 PMC3817493

[brb32809-bib-0071] Mitby, P. A. , Robison, L. L. , Whitton, J. A. , Zevon, M. A. , Gibbs, I. C. , Tersak, J. M. , Meadows, A. T. , Stovall, M. , Zeltzer, L. K. , & Mertens, A. C. (2003). Utilization of special education services and educational attainment among long‐term survivors of childhood cancer: A report from the Childhood Cancer Survivor Study. Cancer, 97(4), 1115–1126. 12569614 10.1002/cncr.11117

[brb32809-bib-0072] Mohrmann, C. , Henry, J. , Hauff, M. , & Hayashi, R. (2015). Neurocognitive outcomes and school performance in solid tumor cancer survivors lacking therapy to the central nervous system. Journal of Personalized Medicine, 5(2), 83–90. 25867598 10.3390/jpm5020083 PMC4493487

[brb32809-bib-0073] Moore, B. D. (2005). Neurocognitive outcomes in survivors of childhood cancer. Journal of Pediatric Psychology, 30(1), 51–63. 15610985 10.1093/jpepsy/jsi016

[brb32809-bib-0074] Moore, B. D. , Copeland, D. R. , & Ried, H. (1992). Neurophysiological basis of cognitive deficits in long‐term survivors of childhood cancer. Archives of Neurology, 49(8), 809–817. 1524513 10.1001/archneur.1992.00530320033009

[brb32809-bib-0075] Mulder, H. , Pitchford, N. J. , & Marlow, N. (2011). Processing speed mediates executive function difficulties in very preterm children in middle childhood. Journal of the International Neuropsychological Society, 17(3), 445–454. 21439114 10.1017/S1355617711000373

[brb32809-bib-0076] Mulhern, R. K. , & Palmer, S. L. (2003). Neurocognitive late effects in pediatric cancer. Current Problems in Cancer, 27(4), 177–197. 12855950 10.1016/s0147-0272(03)00026-6

[brb32809-bib-0077] Mulhern, R. K. , Reddick, W. E. , Palmer, S. L. , Glass, J. O. , Elkin, T. D. , Kun, L. E. , Taylor, J. , Langston, J. , & Gajjar, A. (1999). Neurocognitive deficits in medulloblastoma survivors and white matter loss. Annals of Neurology, 46(6), 834–841. 10589535 10.1002/1531-8249(199912)46:6<834::aid-ana5>3.0.co;2-m

[brb32809-bib-0078] Müller, R. ‐A. (2007). The study of autism as a distributed disorder. Mental Retardation and Developmental Disabilities Research Reviews, 13(1), 85–95. 17326118 10.1002/mrdd.20141 PMC3315379

[brb32809-bib-0079] Murdaugh, D. L. , Maximo, J. O. , & Kana, R. K. (2015). Changes in intrinsic connectivity of the brain's reading network following intervention in children with autism. Human Brain Mapping, 36(8), 2965–2979. 26058572 10.1002/hbm.22821 PMC6869516

[brb32809-bib-0080] Oswald, K. A. , & Bo, J. (2020). Motor functioning and associated cognitive outcomes in pediatric survivors of acute lymphoblastic leukemia. Child Neuropsychology: A Journal on Normal and Abnormal Development in Childhood and Adolescence, 26(5), 597–611. 31594450 10.1080/09297049.2019.1676406

[brb32809-bib-0081] Owens, M. , Stevenson, J. , Norgate, R. , & Hadwin, J. A. (2008). Processing efficiency theory in children: Working memory as a mediator between trait anxiety and academic performance. Anxiety Stress Coping, 21(4), 417–430. 18686056 10.1080/10615800701847823

[brb32809-bib-0082] Palmer, S. L. (2008). Neurodevelopmental impact on children treated for medulloblastoma: A review and proposed conceptual model. Developmental disabilities research reviews, 14(3), 203–210. 18924159 10.1002/ddrr.32 PMC2628167

[brb32809-bib-0083] Palmer, S. L. , Goloubeva, O. , Reddick, W. E. , Glass, J. O. , Gajjar, A. , Kun, L. , Merchant, T. E. , & Mulhern, R. K. (2001). Patterns of intellectual development among survivors of pediatric medulloblastoma: A longitudinal analysis. American Journal of Clinical Oncology, 19(8), 2302–2308. 10.1200/JCO.2001.19.8.2302 11304784

[brb32809-bib-0084] Patel, S. K. , Schulte, F. , Kelly, N. C. , & Steele, A. C. (2016). Neurocognitive late effects in children with cancer. In A. N. Abrams , A. C. Muriel , & L. Wiener (Eds.), Pediatric psychosocial oncology: Textbook for multidisciplinary care (pp. 157–174). Cham, Switzerland: Springer International Publishing; Chapter xix, 409 Pages.

[brb32809-bib-0085] Patel, S. K. , Wong, A. L. , Cuevas, M. , & Van Horn, H. (2013). Parenting stress and neurocognitive late effects in childhood cancer survivors. Psycho‐Oncology, 22(8), 1774–1782. 23097416 10.1002/pon.3213 PMC3721633

[brb32809-bib-0086] Perry, A. , & Schmidt, R. E. (2006). Cancer therapy‐associated CNS neuropathology: An update and review of the literature. Acta Neuropathologica, 111(3), 197–212. 16463065 10.1007/s00401-005-0023-y

[brb32809-bib-0087] Peterson, R. K. , Jones, K. , & Jacobson, L. A. (2021). The contribution of sluggish cognitive tempo to processing speed in survivors of pediatric brain tumors. Child Neuropsychology, 27(7), 960–972. 33866922 10.1080/09297049.2021.1917529 PMC8574989

[brb32809-bib-0088] Phillips, L. H. , Tunstall, M. , & Channon, S. (2007). Exploring the role of working memory in dynamic social cue decoding using dual task methodology. Journal of Nonverbal Behavior, 31(2), 137–152.

[brb32809-bib-0089] Reddick, W. E. , Glass, J. O. , Helton, K. J. , Langston, J. W. , Xiong, X. , Wu, S. , & Pui, C. H. (2005). Prevalence of leukoencephalopathy in children treated for acute lymphoblastic leukemia with high‐dose methotrexate. American Journal of Neuroradiology, 26(5), 1263–1269. 15891195 PMC2396789

[brb32809-bib-0090] Reddick, W. E. , White, H. A. , Glass, J. O. , Wheeler, G. C. , Thompson, S. J. , Gajjar, A. , Leigh, L. , & Mulhern, R. K. (2003). Developmental model relating white matter volume to neurocognitive deficits in pediatric brain tumor survivors. Cancer, 97(10), 2512–2519. 12733151 10.1002/cncr.11355

[brb32809-bib-0091] Reeves, C. B. , Palmer, S. , Gross, A. M. , Simonian, S. J. , Taylor, L. , Willingham, E. , & Mulhern, R. K. (2007). Brief report: Sluggish cognitive tempo among pediatric survivors of acute lymphoblastic leukemia. Journal of Pediatric Psychology, 32(9), 1050–1054. 17933846 10.1093/jpepsy/jsm063

[brb32809-bib-0092] Rijnen, S. J. M. , Meskal, I. , Bakker, M. , De Baene, W. , Rutten, G. ‐J. M. , Gehring, K. , & Sitskoorn, M. M. (2019). Cognitive outcomes in meningioma patients undergoing surgery: Individual changes over time and predictors of late cognitive functioning. Neuro‐Oncology, 21(7), 911–922. 30753679 10.1093/neuonc/noz039 PMC6620637

[brb32809-bib-0093] Robinson, K. E. , Kuttesch, J. F. , Champion, J. E. , Andreotti, C. F. , Hipp, D. W. , Bettis, A. , Barnwell, A. , & Compas, B. E. (2010). A quantitative meta‐analysis of neurocognitive sequelae in survivors of pediatric brain tumors. Pediatric Blood & Cancer, 55(3), 525–531. 20658625 10.1002/pbc.22568

[brb32809-bib-0094] Robinson, K. E. , Pearson, M. M. , Cannistraci, C. J. , Anderson, A. W. , Kuttesch, J. F. , Wymer, K. , Smith, S. E. , & Compas, B. E. (2014). Neuroimaging of executive function in survivors of pediatric brain tumors and healthy controls. Neuropsychology, 28(5), 791–800. 24773415 10.1037/neu0000077

[brb32809-bib-0095] Salami, A. , Eriksson, J. , Nilsson, L. ‐G. , & Nyberg, L. (2012). Age‐related white matter microstructural differences partly mediate age‐related decline in processing speed but not cognition. Biochimica et Biophysica Acta—Molecular Basis of Disease, 1822(3), 408–415. 10.1016/j.bbadis.2011.09.001 21930202

[brb32809-bib-0096] Salthouse, T. A. (1996). The processing‐speed theory of adult age differences in cognition. Published online. 10.1037/0033-295x.103.3.403 8759042

[brb32809-bib-0097] Schatz, J. , Kramer, J. H. , Ablin, A. , & Matthay, K. K. (2000). Processing speed, working memory, and IQ: A developmental model of cognitive deficits following cranial radiation therapy. Neuropsychology, 14(2), 189–200. 10791859 10.1037//0894-4105.14.2.189

[brb32809-bib-0098] Scheuffgen, K. , Happé, F. , Anderson, M. , & Frith, U. (2000). High “intelligence,” low “IQ”? Speed of processing and measured IQ in children with autism. Development and Psychopathology, 12(1), 83–90. 10774597 10.1017/s095457940000105x

[brb32809-bib-0099] Siegwart, V. , Benzing, V. , Spitzhuettl, J. , Schmidt, M. , Grotzer, M. , Steinlin, M. , Leibundgut, K. , Roebers, C. , & Everts, R. (2020). Cognition, psychosocial functioning, and health‐related quality of life among childhood cancer survivors. Neuropsychological Rehabilitation, Published online November 19, 1–24. 10.1080/09602011.2020.1844243 33208044

[brb32809-bib-0100] Sleurs, C. , Deprez, S. , Emsell, L. , Lemiere, J. , & Uyttebroeck, A. (2016). Chemotherapy‐induced neurotoxicity in pediatric solid non‐CNS tumor patients: An update on current state of research and recommended future directions. Critical Reviews in Oncology/Hematology, 103, 37–48. 27233118 10.1016/j.critrevonc.2016.05.001

[brb32809-bib-0101] Smitherman, A. B. , Wood, W. A. , Mitin, N. , Ayer Miller, V. L. , Deal, A. M. , Davis, I. J. , Blatt, J. , Gold, S. H. , & Muss, H. B. (2020). Accelerated aging among childhood, adolescent, and young adult cancer survivors is evidenced by increased expression of p16 ^INK4a^ and frailty. Cancer, 126(22), 4975–4983. 32830315 10.1002/cncr.33112 PMC7607511

[brb32809-bib-0102] Steinlin, M. , Imfeld, S. , Zulauf, P. , Boltshauser, E. , Lövblad, K. O. , Ridolfi Lüthy, A. , Perrig, W. , & Kaufmann, F. (2003). Neuropsychological long‐term sequelae after posterior fossa tumour resection during childhood. A Journal of Neurology, 126(Pt 9), 1998–2008. 10.1093/brain/awg195 12876140

[brb32809-bib-0103] Steliarova‐Foucher, E. , Colombet, M. , Ries, L. A. G. , Moreno, F. , Dolya, A. , Bray, F. , Hesseling, P. , Shin, H. Y. , Stiller, C. A. , & IICC‐3 Contributors . (2017). International incidence of childhood cancer, 2001–10: A population‐based registry study. The Lancet Oncology, 18(6), 719–731. 28410997 10.1016/S1470-2045(17)30186-9 PMC5461370

[brb32809-bib-0104] Tononi, G. (1998). Complexity and coherency: Integrating information in the brain. Trends in Cognitive Sciences, 2(12), 474–484. 21227298 10.1016/s1364-6613(98)01259-5

[brb32809-bib-0105] Torres, V. A. , Ashford, J. M. , Wright, E. , Xu, J. , Zhang, H. , Merchant, T. E. , & Conklin, H. M. (2021). The impact of socioeconomic status (SES) on cognitive outcomes following radiotherapy for pediatric brain tumors: A prospective, longitudinal trial. Neuro‐Oncology, 23, 1173–1182. Published online February 5. 33543269 10.1093/neuonc/noab018 PMC8248851

[brb32809-bib-0106] Ullén, F. (2009). Is activity regulation of late myelination a plastic mechanism in the human nervous system? Neuron Glia Biology, 5(1–2), 29–34. 19785923 10.1017/S1740925X09990330

[brb32809-bib-0107] Van Der Plas, E. , Qiu, W. , Nieman, B. J. , Yasui, Y. , Liu, Q. i. , Dixon, S. B. , Kadan‐Lottick, N. S. , Weldon, C. B. , Weil, B. R. , Jacola, L. M. , Gibson, T. M. , Leisenring, W. , Oeffinger, K. , Hudson, M. M. , Robison, L. L. , Armstrong, G. T. , & Krull, K. R. (2021). Sex‐specific associations between chemotherapy, chronic conditions, and neurocognitive impairment in acute lymphoblastic leukemia survivors: A report from the childhood cancer survivor study. JNCI: Journal of the National Cancer Institute, 113(5), 588–596. 32882041 10.1093/jnci/djaa136 PMC8096369

[brb32809-bib-0108] Vasa, R. A. , Mostofsky, S. H. , & Ewen, J. B. (2016). The disrupted connectivity hypothesis of autism spectrum disorders: Time for the next phase in research. Biological Psychiatry: Cognitive Neuroscience and Neuroimaging, 1(3), 245–252. 28083565 10.1016/j.bpsc.2016.02.003 PMC5222574

[brb32809-bib-0109] Waber, D. P. , Urion, D. K. , Tarbell, N. J. , Niemeyer, C. , Gelber, R. , & Sallan, S. E. (1990). Late effects of central nervous system treatment of acute lymphoblastic leukemia in childhood are sex‐dependent. Developmental Medicine and Child Neurology, 32(3), 238–248. 2311827 10.1111/j.1469-8749.1990.tb16930.x

[brb32809-bib-0110] Wechsler, D. (2008). WAIS‐IV: Technical and interpretive manual. The Psychological Corporation.

[brb32809-bib-0111] Wechsler, D. (2014). WISC‐V: Technical and interpretive manual. Pearson.

[brb32809-bib-0112] Weiss, L. G. , Saklofske, D. H. , Holdnack, J. A. , & Prifitera, A. (2016). WISC‐V assessment and interpretation: Scientist practitioner perspectives. Elsevier.

[brb32809-bib-0113] Willard, V. W. , Hardy, K. K. , Allen, T. M. , Hwang, E. I. , Gururangan, S. , Hostetter, S. A. , & Bonner, M. J. (2013). Sluggish cognitive tempo in survivors of pediatric brain tumors. Journal of Neuro‐Oncology, 114(1), 71–78. 23661102 10.1007/s11060-013-1149-8

[brb32809-bib-0114] Williams, A. M. , & Cole, P. D. (2021). Biomarkers of cognitive impairment in pediatric cancer survivors. Journal of Clinical Oncology, 39(16), 1766. 33886369 10.1200/JCO.20.02436

[brb32809-bib-0115] Williams, A. M. , Krull, K. R. , Howell, C. R. , Banerjee, P. , Brinkman, T. M. , Kaste, S. C. , Partin, R. E. , Srivastava, D. , Yasui, Y. , Armstrong, G. T. , Robison, L. L. , Hudson, M. M. , & Ness, K. K. (2021). Physiologic frailty and neurocognitive decline among young‐adult childhood cancer survivors: A prospective study from the St. Jude lifetime cohort. Journal of Clinical Oncology, 39(31), 3485–3495. 34283634 10.1200/JCO.21.00194 PMC8547937

[brb32809-bib-0116] Winter, A. L. , Conklin, H. M. , Tyc, V. L. , Stancel, H. , Hinds, P. S. , Hudson, M. M. , & Kahalley, L. S. (2014). Executive function late effects in survivors of pediatric brain tumors and acute lymphoblastic leukemia. Journal of Clinical and Experimental Neuropsychology, 36(8), 818–830. 25126830 10.1080/13803395.2014.943695 PMC4229447

[brb32809-bib-0117] Wolfe, K. R. , Madan‐Swain, A. , & Kana, R. K. (2012). Executive dysfunction in pediatric posterior fossa tumor survivors: A systematic literature review of neurocognitive deficits and interventions. Developmental Neuropsychology, 37(2), 153–175. 22339228 10.1080/87565641.2011.632462 PMC3730812

[brb32809-bib-0118] Wu, T. C. , Wilde, E. A. , Bigler, E. D. , Li, X. , Merkley, T. L. , Yallampalli, R. , Mccauley, S. R. , Schnelle, K. P. , Vasquez, A. C. , Chu, Z. , Hanten, G. , Hunter, J. V. , & Levin, H. S. (2010). Longitudinal changes in the corpus callosum following pediatric traumatic brain injury. Developmental Neuroscience, 32(5‐6), 361–373. 20948181 10.1159/000317058 PMC3073757

[brb32809-bib-0119] Yuan, W. , Treble‐Barna, A. , Sohlberg, M. M. , Harn, B. , & Wade, S. L. (2017). Changes in structural connectivity following a cognitive intervention in children with traumatic brain injury: A pilot study. Neurorehabilitation and Neural Repair, 31(2), 190–201. 27798379 10.1177/1545968316675430

